# 

**Published:** 1850-11

**Authors:** 


### CONTENTS:

### PART I.—ORIGINAL COMMUNICATIONS.

Davis' *Medical* History, - - - - - - - p.367

ART. 1.—Electro Galvanism—By Thos. D. Washburn, M.*D.* - 288

ART. 2,—Congestive Fever—By E. S. Cooper, M.*D.* - - - 299

ART. 3.—Treatment of Cholera—By B. F. Mullen. M.*D.* - . 306

ART. 4.—Case of Absence of the Uterus—By Geo. Smith Crawford, M.D, 308

ART. 5.—Remarkable change of position of the Spleen and Abscess of the same—By J. J. Lescher, M.D. - - - - - 309

ART. 6.—Two Fœtuses of unequal size at the same Abortion—By Dr. F. Brady, - - - - - - - - - 314

ART. 7.—Treatment of Rubeola by Inunction—By John Evans, M.D. 315

ART. 8.—Tracheotomy for Abscess in the root of the Tongne, resulting successfully—By Daniel Brainard, M.D. - - - - 316

ART. 9.—Poisoning by Stramonium—By Ira E. Oatman, M.D. - 317

### PART II.—REVIEWS AND NOTICES OF NEW WORKS.

ART. 1.—Medical Jurisprudence—By Alfred Taylor, M.D. - - 319

ART. 2.—A Theoretical and Practical Treatise on Midwifery including the Diseases of Pregnancy and Parturition—By P, Cazeau, 320

ART. 3.—General Therapeutics and Materia Medica; adapted for a Medical Text Book—By Robly Dunglison, M.D. - - - 321

ART. 4.—History of Medical Education and Institutions in the United States, etc.—By N. S. Davis, M.D. - - - - 322

ART. 5.—Essays on Puerperal Fever and the diseases peculiar to women, selected from the writings of British Authors previous to the close of the eighteenth century—Edited by Fleetwood Churchill, M.D. 323

ART. 6.—On the Use and Abuse of Alcoholic Liquors, in Health and Disease—By William B. Carpenter, M.D. - - - - 324

ART. 7.—A practical Treatise on Inflammation, &c., of the Uterus and its appendages—By James Henry Bennett, M.D. - - - 328

### PART III.—SELECTIONS.

ART. 1.—Cases of Asthma successfully treated by Nitric Acid—By T. S. Hopkins, M.D. - - - - - - - - 329

ART. 2.—New Adhesive Dressing—By Dr. Mellez, - - - - 331

### PART IV.—EDITORIAL.

ART. 1.—Maclise's Surgical Anatomy. - - - 333

ART, 2.—Reese's Formulary, - - - - - - 333

ART. 3.—The *Dentists*, - - - - - - 334

ARR. 4.—Transactioos of the Am, Med, Association, Vol, 3, - - 334

ART. 5.—Editorial Etiquette, - - - - - - 335

ART. 6.—Tilden's Extracts, - - - - - 336

ART. 7.—New Medical Journals, - - - - 336

ART. 8,—Homœopathic Impertinence, - - - - 337

ART. 9.—Miscellaneous Medical Intelligence, - - - - 338

